# Supramolecular Ionic Gels for Stretchable Electronics and Future Directions

**DOI:** 10.1021/acsmaterialsau.4c00100

**Published:** 2024-11-22

**Authors:** Shunsuke Yamada, Takashi Honda

**Affiliations:** Department of Electrical and Electronic Engineering, Kyushu Institute of Technology, 1-1 Sensuicho, Tobataku, Kitakyushu, Fukuoka 804-8550, Japan

**Keywords:** supramolecular ionic gel, ionic liquid, wearables, stretchable electronics, self-healing

## Abstract

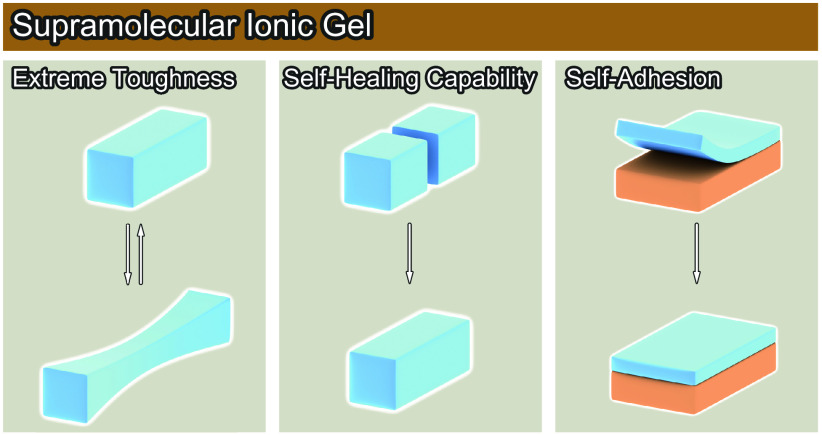

Ionic gels (IGs), ionic liquids (ILs) dispersed in polymers, exhibit extremely low vapor pressure, electrochemical and thermal stability, and excellent mechanical characteristics; therefore, they are used for fabricating stretchable sensors, electrochemical transistors, and energy storage devices. Although such characteristics are promising for flexible and stretchable electronics, the mechanical stress-induced ruptured covalent bonds forming polymer networks cannot recover owing to the irreversible interaction between the bonds. Physical cross-linking via noncovalent bonds enables the interaction of polymers and ILs to form supramolecular IGs (SIGs), which exhibit favorable characteristics for wearable devices that conventional IGs with noncovalent bonds cannot achieve. Herein, we review recent material designs and interactions used for fabricating SIGs, such as ionic interactions and hydrogen bonding. We present SIG characteristics achieved via the interaction of polymers and ILs, such as extreme toughness, self-healing capability, and self-adhesion favorable for human body sensors. We conclude this Perspective by discussing the potential of SIGs as a power source for implants, wearable devices, and environmental sensing applications.

## 1 Introduction

**Figure 1:**
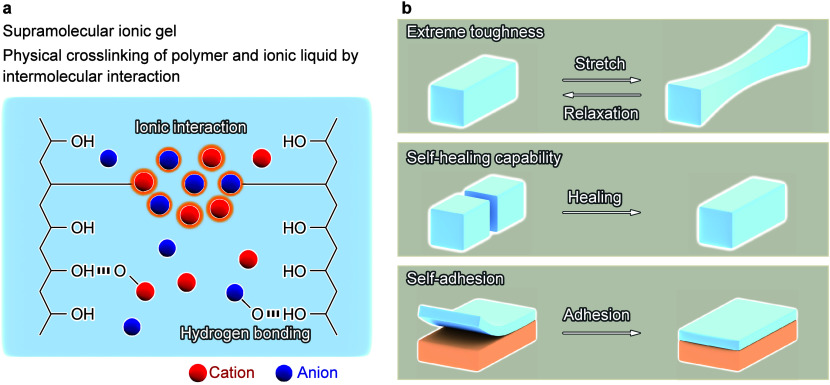
Schematic of the intermolecular interaction of SIGs and their characteristics. (a) Physical cross-linking via ionic interactions and hydrogen bonds. (b) SIG physical properties: extreme toughness, self-healing capability, and self-adhesion associated with reversible reactions via physical cross-linking.

Electronic skin comprising soft materials has been a research area in electronics, mechanical engineering, and materials science owing to its high affinity to human skin for biomedical applications.^1−3^ Organic semiconductors^4^ comprising intrinsically soft materials are used to develop stretchable transistors,^5^ sensors,^6^ and radio frequency circuits.^7^ The integrated electronic skin acquires information from the human body for health monitoring.^8^ Ionic liquids (ILs) are used in flexible and stretchable devices as sensing materials owing to their extremely low vapor pressure,^9^ high ionic conductivity,^10,11^ and electrochemical^12^ and thermal stability.^13−15^ ILs disperse into polymer networks to produce solid-state electrolytes, ionic gels (IGs),^16−18^ which improve their handling properties. Photo-cross-linking is a facile and established process to produce IGs by dissolving monomers and photoinitiators in ILs, followed by ultraviolet curing.^19^ IGs exhibit low Young’s modulus and high stretchability, maintaining favorable characteristics of ILs.^20^ However, covalent bond formation by chemical cross-linking is an irreversible reaction and cannot heal IGs ruptured under harsh mechanical stress caused by human motion. Multiple molecular complexes formed via the physical cross-linking of supramolecular polymers with dynamic noncovalent bonds exhibit characteristics such as toughness,^21^ self-healing capability,^22,23^ and stretchability,^24^ which are unachievable using conventional polymers with covalent bonds. Inspired by supramolecular networks, the reversible intermolecular interactions of ILs with polymers were used to develop supramolecular IGs (SIGs) with promising mechanical characteristics for electronic skin (Figure 1a).

In this perspective, we review the recent progress of SIGs. We focus on their characteristics, IL–polymer interactions, and electronic skin applications. The mechanical characteristics of SIGs such as their extremely high mechanical robustness, self-healing capability, and self-adhesion (Figure 1b) are highlighted. The applications of SIGs for stretchable devices with self-healing capability and self-adhesion for wearable devices are also reviewed. Finally, this perspective concludes with an outlook to expand the SIG uses for practical wearable devices and environmental sensing applications.

## 2 Characteristics of SIGs

### 2.1 Mechanical Robustness

**Figure 2:**
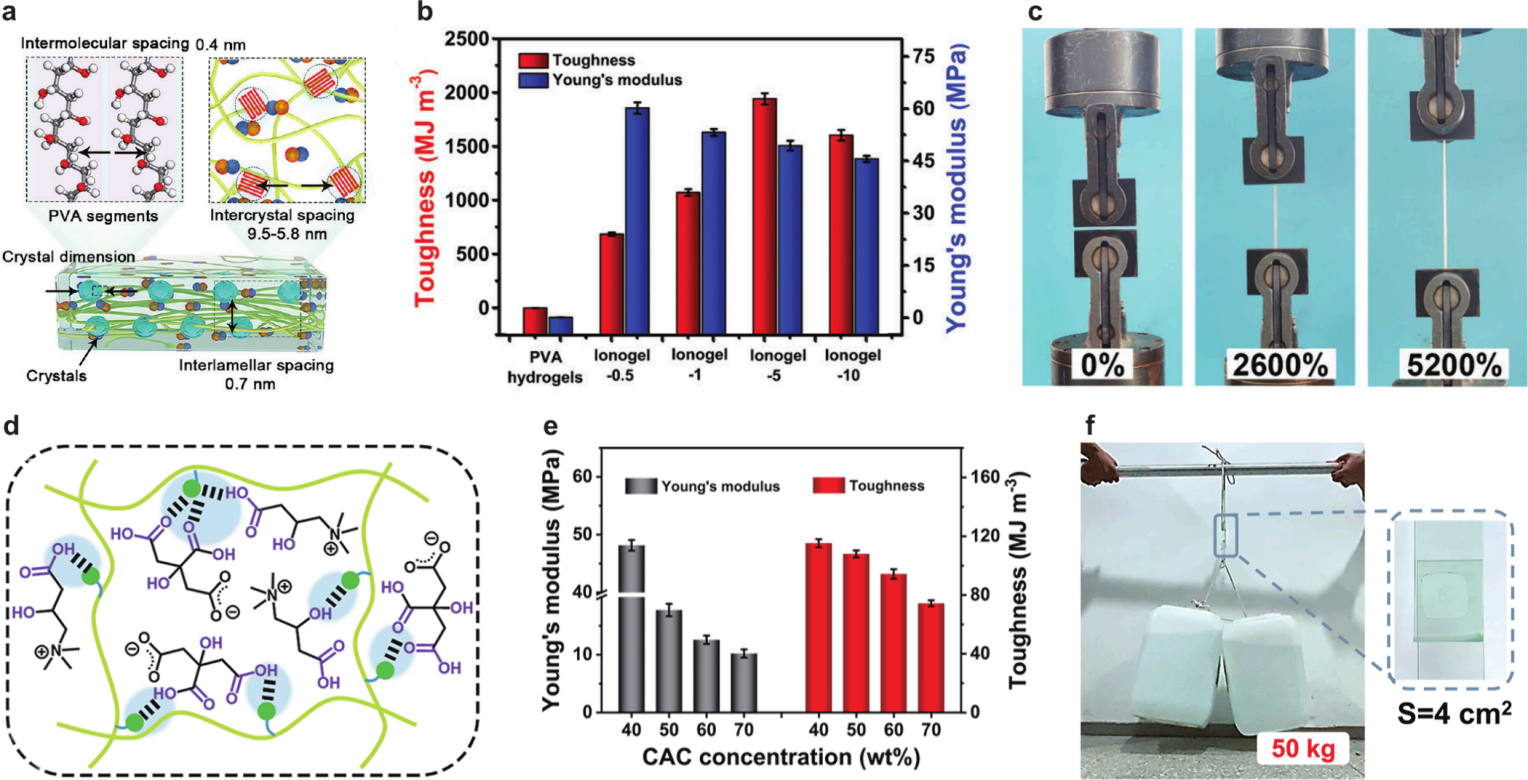
Extreme toughness of SIGs. (a) Schematic of nanocrystalline structures in a SIG comprising PVA and IL. (b) Toughness and Young’s modulus with various IL loadings. (c) Photograph of an SIG under a tensile stress test, reproduced with permission from ref (25). Copyright 2023 Wiley-VCH. (d) Schematic of SIG-incorporated poly *N*,*N*-dimethylacrylamide and CAC as an IL. (e) Toughness and Young’s modulus with various IL concentrations. (f) Demonstration of SIG adhesion to carry 50 kg buckets, reproduced from ref (26). Copyright 2024 American Chemical Society.

Flexible and stretchable electronics adopt IGs as sensing components for tactile or strain sensors. IGs are subjected to repetitive mechanical strain, stress, and pressure. Thus, the mechanical robustness of IGs is essential for long-term operation. Li et al. developed unprecedentedly robust SIGs comprising poly(vinyl alcohol) [PVA] and a halometallate IL 1-butyl-3-methylimidazolium zinc bromide ([Bmim][Zn_*x*_Br_*y*_]).^25^ The metal-containing anionic group of the IL can form hydrogen bonds with the hydroxyl (−OH) groups of PVA and the imidazolium ring (Figure 2a). The Fourier-transform infrared spectra of PVA exhibited two intense peaks at 3292 and 1086 cm^–1^ attributed to the stretching vibrations of–OH and C–O, respectively. The addition of IL to PVA shifted the peaks to 3340 and 1074 cm^–1^, respectively, because of hydrogen bonding and coordination between PVA and [Bmim][Zn_*x*_Br_*y*_]. Molecular interaction shifted the peaks ascribed to C–H stretching vibration from 3106 and 3148 cm^–1^ to 3110 and 3144 cm^–1^, respectively, indicating the presence of hydrogen bonds between PVA hydroxyl groups and imidazolium cations. PVA nanocrystalline structures were formed in IGs under strain. In situ wide-angle X-ray scattering (WAXS) and small-angle X-ray scattering exhibited two intense peaks in the WAXS one-dimension patterns at *d*-spacings of 0.4 nm (average distance between the adjacent PVA segments in the nanocrystalline microregions) and 0.7 nm (average layer spacing between nanocrystalline domains perpendicular to the loading direction), respectively. The initial average spacing between the adjacent nanocrystalline domains of the ionogel was 9.5 nm, while the corresponding average spacing decreased to 8.6, 7.2, and 5.8 nm when the strain values on the IG were 500%, 1000%, and 1500%, respectively. Thus, an increase in the external load strain produced nanocrystalline structures in IGs. Furthermore, two-dimensional WAXS patterns indicate the enhancement of intensity associated with the crystalline scattering of PVA with 0.4 nm *d*-spacing with the increase in loading strain, confirming that additional amorphous chain segments are converted to nanocrystalline microregions. The increase in nanocrystalline microregions under strain strengthens the progressive crystallization mechanism, affording IGs with ultrahigh strength and fracture toughness. SIGs were termed ionogel-*x* (ionogel-0.5, −1, −5, and −10), where *x* represents the molar ratio of [Bmim]Br and ZnBr_2_ in the halometallate IL used for the synthesis of SIGs. Ionogel-0.5, −1, −5, and −10 exhibited extremely high ultimate fracture stresses of 37.5 ± 1.3, 53.5 ± 1.7, 63.1 ± 2.1, and 52.8 ± 1.2 MPa and ultimate fracture strains of 2416% ± 75%, 3440% ± 95%, 5248% ± 113%, and 5100% ± 126%, respectively. The toughness values of ionogel-0.5, −1, −5, and −10 were 690 ± 16, 1077 ± 32, 1947 ± 52, and 1610 ± 49 MJ m^–3^ with corresponding Young’s modulus of 60.3 ± 1.6, 53.3 ± 1.0, 49.5 ± 1.4, and 45.7 ± 0.9 MPa (Figure 2b). The toughness and Young’s modulus of PVA hydrogels were 3.77 ± 0.25 MJ m^–3^ and 0.17 ± 0.01 MPa, respectively. SIGs exhibit better mechanical characteristics due to their progressive crystallization strengthening mechanism compared to those of PVA hydrogels. In addition, SIGs can stretch up to 5200% and are promising for stretchable electronic applications (Figure 2c).

SIG-based wearable devices should be in contact with the human skin to retrieve human body information. Hence, the biocompatibility of SIGs is essential to make a safe human–machine interface. Xiong et al. developed bioapplicable SIGs using a citric acid/*L*-(−)-carnitine (CAC)-based IL and poly(dimethyl acrylamide) as an electrolyte and polymer (Figure 2d).^26^ Citric acid (CA) and *L*-(−)-carnitine (LC) are recognized as safe materials. CA is used in food, pharmaceutical, and cosmetic industries, while LC is a water-soluble molecule that regulates metabolism. As CAC exhibits high biocompatibility, SIG comprising CAC also exhibits the same. The –COOH of LC and CA forms hydrogen bonding with linear polymer segments, forming reversible physical cross-linking that induces high strength and toughness, insensitivity to crack extension, self-healing, and recyclability to SIGs. SIGs with 70 wt % CAC deformed up to 49-fold longer than their initial length and retained a tensile strength of 4.1 MPa. A decrease in the CAC content reduced the segment strain capacity and enhanced the SIG strength. SIGs with 40 wt % CAC exhibited the maximum Young’s modulus and toughness of 48 MPa and 115 MJ m^–3^, respectively (Figure 2e). Apart from extreme toughness, SIGs exhibited rigid adhesion by attaching two glass sheets with 4 cm^2^ dimension, which lifted 50-kg buckets (Figure 2f).

### 2.2 Self-Healing Capability

**Figure 3:**
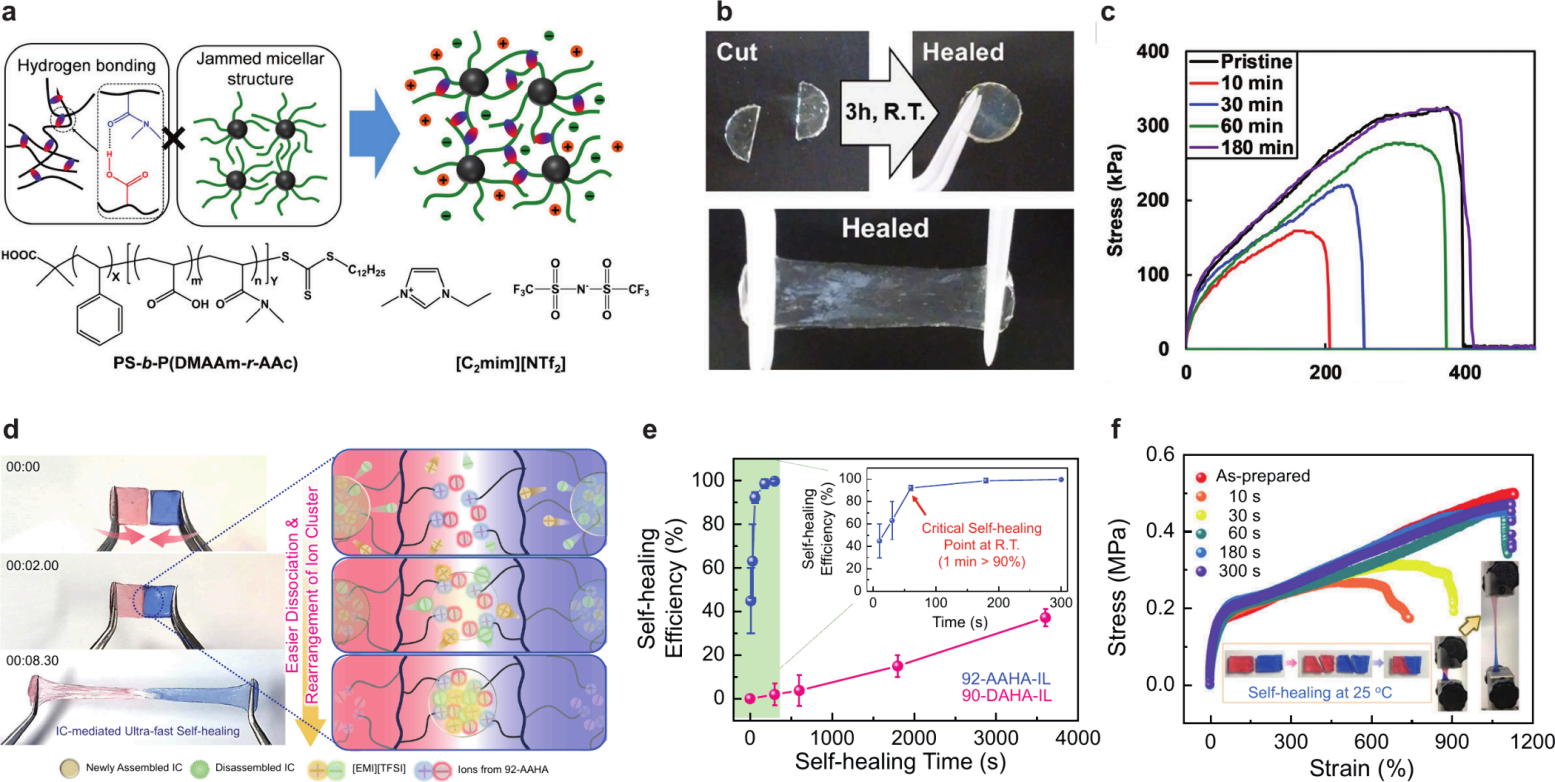
SIG self-healing capability and characteristics. (a) Schematic of an SIG comprising a block copolymer and an IL [C_2_mim][NTf_2_] with its nanostructures formed via hydrogen bonding and IL-phobicity. (b) Photographs of a cut and self-healed SIG after 3 h. (c) Tensile stress curves with different self-healing durations, reprinted with permission from ref (27). Copyright 2022 Wiley-VCH. (d) Photograph of the self-healing behavior of SIGs and the schematic mechanism associated with formation/dissociation of ion clusters. (e) Comparison of the self-healing efficiency using an ionic and a nonionic polymer. (f) Tensile stress curves with different self-healing times, reproduced from ref (28). Available under a CC-BY 4.0 license http://creativecommons.org/licenses/by/4.0/. Copyright 2018 Kim et al.

Although SIGs possess high toughness, they rupture owing to repetitive mechanical strain or stress. Thereby, self-healing capability is desired to recover ionic and mechanical characteristics after SIG rupture. Tamate et al. synthesized diblock copolymers comprising polystyrene (PS), *N*,*N*-dimethylacrylamide (DMAAm), and acrylic acid (AAc) [PS-*b*-P(DMAAm-*r*-AAc)] for SIG self-healing (Figure 3a).^27^ The hydrogen bonding block (DMAAm and AAc) physically cross-links reversibly to achieve self-healing capability. The IL-phobic block (PS) suppresses terminal flow behavior associated with the transient physical cross-linking of hydrogen bonds to yield a free-standing gel with the micellar structure of the PS block. Polymers with solid hydrogen bonds show poor solubility in ILs. Hence, polymer designs containing hydrogen bond acceptors and donors allow the synthesis of IGs with a self-healing capability via hydrogen bond formation. An IL, ethyl-3-methylimidazolium bis(trifluoromethanesulfonyl)amide ([C_2_mim][NTf_2_]) could disperse into the copolymer P(DMAAm-*r*-AAc) with a [DMAAm]:[AAc] ratio of 8:2 to form a transparent IG without phase separation. The presence of [DMAAm] < 7 in the ratio induced phase separation to produce an opaque and brittle gel owing to the strong hydrogen bonds of polymer chains. The diblock copolymer PS-*b*-P(DMAAm-*r*-AAc) with the [DMAAm]:[AAc] ratio of 8:2 yielded a self-healing IG with a copolymer weight ratio of 30 wt %. Disk-shaped IGs cut into two fragments could be stretched by tweezers for 3 h after healing at room temperature (Figure 3b). Tensile stress tests revealed that pristine IGs showed fracture stress and strain of 0.32 MPa and 400%, (Figure 3c). Healing after 10 min recovered the IGs and they exhibited fracture stress and strain of 0.15 MPa and 200%, respectively. IGs healing after 180 min exhibited a stress and strain curve nearly similar to that of pristine IGs. Moreover, the IGs exhibited a high ionic conductivity of up to 1.2 mS cm^–1^ at 30 °C.

Kim et al. developed self-healing IGs with a rapid dynamic formation–dissociation of ion clusters using poly([(3-acryloamidopropyl)-trimethylammonium] [bis(trifluoromethanesulfonyl) imide])-*ran*-poly(2-hydroxyethyl acrylate) (P[AA][TFSI]-*r*-PHEA, AAHA) and a room temperature IL 1-ethyl-3-methylimidazolium bis(trifluoromethylsulfonyl)imide ([EMI][TFSI]) as a polymer network and electrolyte, respectively (Figure 3d).^28^ The AAHA copolymer comprises charged pendant groups (P[AA][TFSI]) and hydrogen bonding sites (PHEA). Apart from hydrogen bonding, the electrostatic interaction of [EMI][TFSI] with P[AA][TFSI] in the copolymer forms supramolecular networks using noncovalently bonded ionic clusters. The [EMI][TFSI] content in the two ionoconductors was fixed at 30 wt %. Tensile stress tests exhibited that the as-prepared 92-AAHA-IL with a mole fraction of 8% hydroxyl groups is elastic, exhibiting stretchability up to ∼1130% and excellent mechanical stability. 92-AAHA-IL dissociated into two fragments self-healed to regain ∼93.1% and ∼96.6% of the original yield strength and stretchability after 60 s. Furthermore, the time-dependent self-healing efficiencies were evaluated based on the toughness ratio between the repaired and original ionic conductors. Healing for 1 min at 25 °C enables 92-AAHA-IL to recover its original mechanical properties with a high efficiency of ∼90.3%. Repair times of 3 and 5 min exhibited efficiencies of ∼98.7% and ∼99.7%, respectively (Figure 3f). The long-term performance of SIGs under sustained mechanical stress is essential for the stable operation of wearables exposed to harsh mechanical conditions. Cyclic uniaxial tensile tests revealed that a SIG sensor exhibits a relative resistance (Δ*R*/*R*_0_) of 60.2%, the value of which remains unchanged over the initial 6 000 cycles. After the SIG was cut into two pieces and allowed to self-heal, the *ΔR*/*R*_0_ remained stable over an additional 6 000 cycles. The influence of self-healing behavior on the relation in Δ*R*/*R*_0_ and applied strain was evaluated. The slope of a linear fit of Δ*R*/*R*_0_ with respect to strain provides the SIG gauge factor, which showed no significant change even after 50 cycles. After a SIG was cut into two pieces and aged for 6 months, it could self-heal due to IL’s extremely low vapor pressure. This stable, long-term self-healing capability promises wearable devices subjected to repetitive stretching.

### 2.3 Self-Adhesion

**Figure 4:**
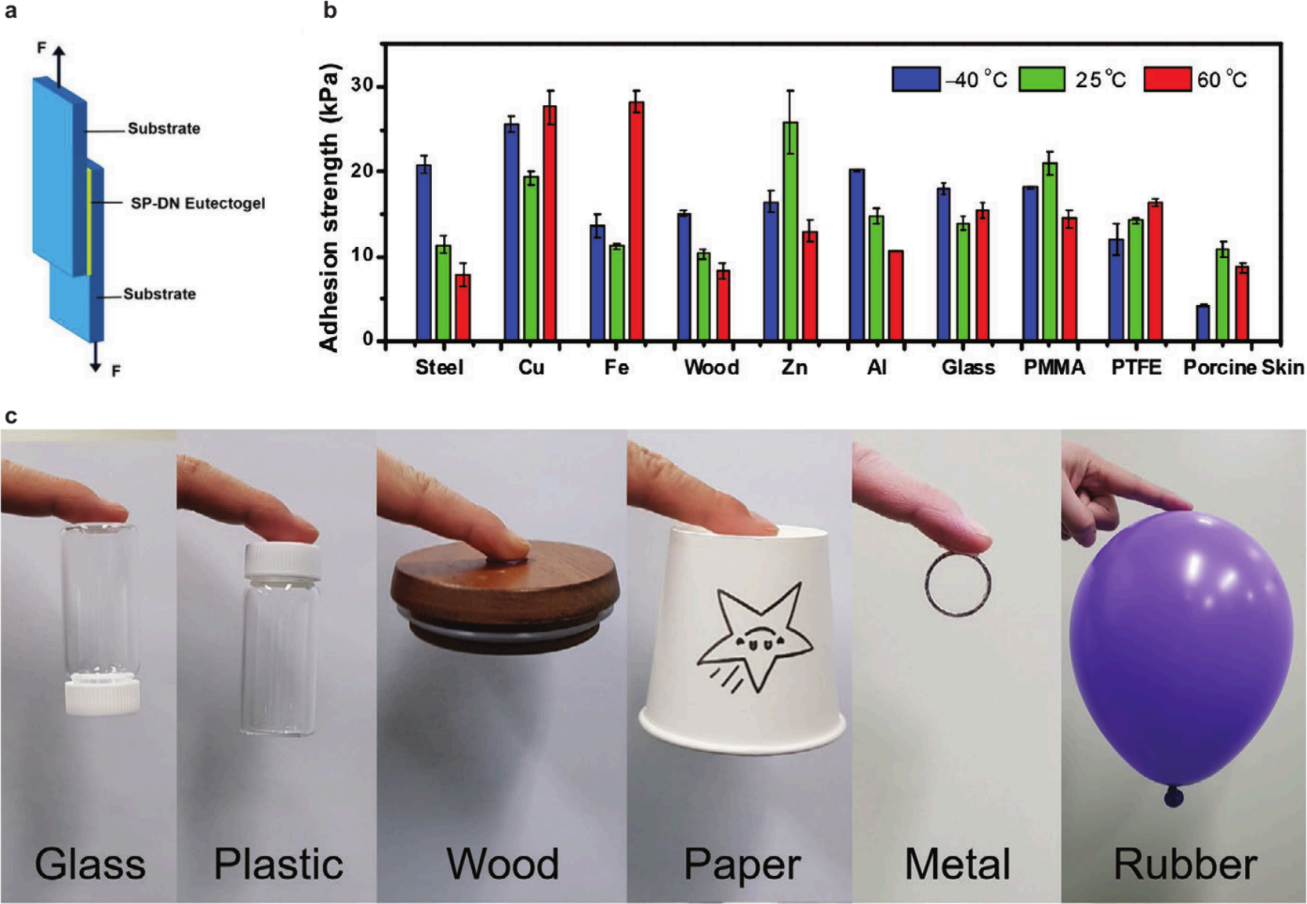
SIG self-adhesion strength and its demonstration. (a) Experimental setup of lap shear tests to evaluate SIG adhesion strength and (b) results with different materials at −40, 25, and 60 °C, reproduced with permission from ref (29). Copyright 2021 Wiley-VCH. (c) Photographs of SIGs adhering to various organic and inorganic materials, reproduced with permission from ref (30). Copyright 2021 Wiley-VCH.

The self-adhesion of SIGs is a crucial feature for flexible and stretchable electronics for their use at the human–machine interface. Liang et al. demonstrated a low-molecular weight supramolecular polymer double network (SPDN) strategy to formulate eutectogel with in-built self-adhesion.^29^ The SPDN formed an amphiphilic low-molecular weight gelator with (*R*)-12-hydroxystearic acid hydrazide (HSAH), poly(*N*-hydroxyethyl acrylamide) (PHEAA), choline chloride:propane-1,3-diol (ChCl:PDO = 1:2) as the first and second polymer networks, and electrolyte, respectively. The SPDN eutectogels (HSAH/PHEAA) exhibited high stretchability of >4000% elongation. In addition, lap shear tests showed that SPDN eutectogels exhibited strong adhesion to various inorganic (stainless steel, copper, iron, zinc, and aluminum) and organic (polytetrafluoroethylene [PTFE] and poly(methyl methacrylate) [PMMA]) materials, and porous wood (Figures 4a and 4b). HSAH/PHEAA exhibited excellent adhesion strength of 13.92 ± 0.77 kPa, 14.75 ± 0.89 kPa, and 14.23 ± 0.32 kPa to glass, aluminum, and PTFE plates, respectively. Moreover, HSAH/PHEAA also exhibited superb adhesion strength to human skin and porcine skin surfaces (10.90 ± 0.97 kPa). The adhesion was intact over a wide temperature range of from –40 to 60 °C.

Cho et al. developed SIGs comprising an associated polymer network of two block copolymers (BCPs) in 1-ethyl-3-methylimidazolium bis(trifluoromethyl sulfonyl)imide ([EMI] [TFSI]).^30^ The two BCPs comprised an IL-phobic poly(*tert*-butylstyrene) and IL-philic poly(methyl acrylate-random-4-hydroxystyrene) [poly(MA-*r*-4HS)] or poly(methyl acrylate-random-2-vinylpyridine) [poly(MA-*r*-2VP)] blocks. The IL dissolved in BCPs segregated poly(*tert*-butylstyrene) blocks to form micellar clusters, whereas the 4HS units or 2VP units adhered to each other through interchain of hydrogen bonds. The functional groups (2VP, 4HS, and ester units) in the polymer network interacted with various materials exhibiting strong adhesion. The concentration of the two BCPs in associated polymer network–based SIGs was maintained at 30 wt %. SIGs with weight ratios of PSHM:PSVM of 3:1, 2:1, 1:1, 1:2, and 1:3 were denoted as HV31, HV21, HV11, HV12, and HV13, respectively. Shear and lift-off force via gel adhesion was evaluated by removing gels attached to substrates at 90° or 180°. The adhesive strength of HV31, HV21, and HV11 gels to a polyimide (PI) film was weak (∼1 kPa). However, HV12 or HV13 required a relatively high force to separate the PI film. The highest shear stress and lift-off force of HV12 were 57.8 kPa and 93.3 N m^–1^, respectively. Thus, the free 2VP unit is beneficial for the strong adhesion of the associated polymer network–based SIG system because the phenol units in HV31, HV21, and HV11 can form hydrogen bonds with another phenol group, reducing the number of effective sites for adhesion. HV12 adhered to various materials such as Au and Al metals, PI and poly(ethylene terephthalate) plastics, and PDMS and Ecoflex rubber samples (Figure 4c). The adhesion ability of HV12 was examined by placing HV12 on a finger and examining its adhering capability. The finger adhered to a variety of materials such as glass, plastic, wood, paper, metal (silver ring), and rubber (balloon). A sensor employing the SIG showed the stable electrical signal upon repetitive cycles at 100% strain for 450 cycles.

### 2.4 SIG Application for Wearable Devices

**Figure 5:**
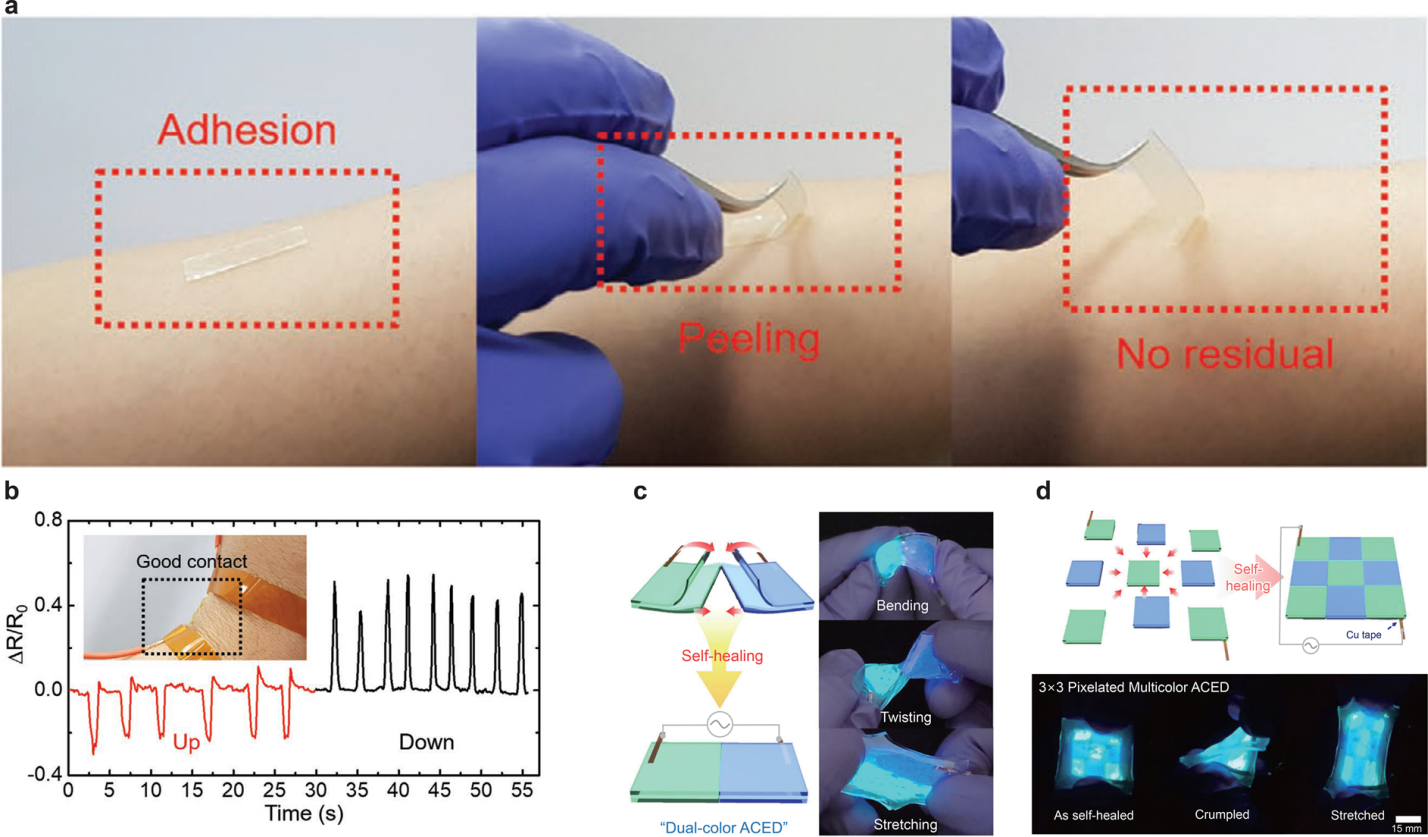
SIG applications for a sensor and display. (a) SIG adhesion to human skin and (b) sensing signals with self-adhesive SIG, reproduced with permission from ref (30). Copyright 2021 Wiley-VCH. (c) Photograph of the self-healing display of bending, twisting, and stretching. (d) A display with 3 × 3 pixels assembled via self-healing green and blue cells, reproduced from ref (28). Available under a CC-BY 4.0 license http://creativecommons.org/licenses/by/4.0/. Copyright 2018 Kim et al.

The aforementioned physical properties are promising to develop devices that attach to the human skin for biomedical applications. Cho et al. developed strain sensors that could precisely detect human motion owing to their strong adhesion to human skin (Figure 5a).^30^ A strain sensor with a conventional gel gets detached from the human skin during the bending of the wrist. Alternately, the SIG-based strain sensor maintained skin contact and exhibited a sharp and intense change of resistance *ΔR*/*R*_0_ to the wrist bending up and down (Figures 5b and 5c). Apart from the wrist motion, the sensor can distinguish the bending angle of a singer, walking, running, jumping, and the three-way movements of the neck combining stretching and compressing. Liang et al. also developed strain sensors using SIGs. In addition to the excellent adhesion to human skin, the strain sensor exhibited stable strain sensitivity from below subzero temperatures (−40 °C) to high temperatures (60 °C) owing to the remarkable antifreezing and solvent-retention capacities of gels.^29^ SIGs exhibit self-healing capability, allowing the development of sensors with stable and long-term operation under harsh mechanical stress owing to recovery after rupturing.

SIGs are transparent ionic conductors and promising candidates for transparent electrodes for displays. Kim et al. developed an SIG-based alternating current electroluminescent display (ACED) using 92-AAHA IL and ZnS:Cu, or ZnS:Cu and Al as electrodes and an emissive layer (green or blue), respectively (Figure 5c).^28^ The application of a symmetric square wave (with a peak-to-peak voltage of 200 V_pp_ at 25 kHz) emits a green light from the ZnS:Cu display. The device was cut into two components using a razor blade. The two individual devices were attached along the cross-section to be healed. Although the emissive layer did not have self-healing ability, the resulting ACED self-healed both electrodes and operated similarly to the original electrode. The self-healing capability of SIGs enabled the fabrication of a dual-color emitting device combining green and blue emitting units (Figure 5d). The combination of the cells into a single device allows blue and green cells to operate independently without noticeable interference. The combined device exhibited high deformation capability, allowing operation under bending, twisting, and stretching. The assembly was scaled to produce an ACED with 3 × 3 pixels of five green and four blue units. All cells were illuminated simultaneously after supplying power. The 3 × 3 pixelated multicolor ACED exhibited excellent deformation even when crumpled and stretched.

## 3 Summary and Outlook

Recent progress in materials science has developed SIGs with excellent toughness, self-healing capability, and self-adhesion owing to the physical cross-linking of polymers and ILs. Table 1 summarized the comparison of materials, maximum fracture stress, strain, Young’s modulus, adhesion strength, and self-healing efficiency of the SIGs. SIGs have been used to develop stretchable and mechanically robust devices suitable for wearable devices. SIG Young’s modulus and stretchability must closely match those of human skin to make SIG wearables comfortable to wear on the skin. Human tissues typically exhibit Young’s moduli at the kilopascal (kPa) level, for example, skin (∼420–850 kPa),^31^ a blood vessel (∼430–870 kPa),^32^ and muscle (medial gastrocnemius muscle, ∼ 15–30 kPa; lateral gastrocnemius muscle, ∼15–30 kPa; Achilles tendon, ∼250–500 kPa).^33^ As for the stretchability, the SIGs accommodate strains of around 30% at skins^34^ and over 100% at joints.^35^ SIG Electrochemical properties are essential for applications in sensing, energy storage, and bioelectrode. Integrated circuit (IC) chips and radio frequency (RF) circuits have operating voltage of at least 1.8 V^36^ and 2.0 V,^37^ respectively. Thus, SIGs potential windows should surpass those values to develop energy storage devices such as supercapacitors and batteries. High ionic conductivity is beneficial for SIG sensors, electrodes, and bioelectrodes, as it yields low signal-to-noise ratios, accurately capturing human body information.^38^ ILs are hygroscopic and absorbs moisture air, resulting in variation of the electrochemical properties. Thereby, adopting hydrophobic IL and polymer are crucial, achieving stable and long-term operation of SIG devices. The recent applications of electrochemical devices with SIGs includes strain sensors, displays, and supercapacitors. These devices are not limited to single-cell configurations but instead utilize large-scale, multiarray designs for multipoint sensing, image display, and enhanced capacity. Consequently, development routine is crucial for scaling up the SIG devices. SIG precursors, which are liquid formulations containing ionic liquids, polymers, and solvents, facilitate the production of printable inks compatible with conventional printing techniques, including 3D printing,^39^ inkjet printing,^40^ and direct ink wiring.^41,42^ The extreme toughness, self-healing capability, and self-adhesion of SIGs are attributed to the intermolecular interactions between ionic liquids and polymers; these printing methods allow SIG devices to scale up without compromising these properties.^42^ Furthermore, these printing techniques enable the creation of large-scale SIG patterns, crucial for designing various devices, including electrochemical transistors,^43−45^ bioelectrodes,^46−48^ and secondary batteries.^49−52^ SIG-based wearables must be attached directly to human skin to capture physiological data, making biocompatibility a critical requirement for safe human-machine interfaces. Niu et al. developed biocompatible supramolecular hydrogel using PVA and bioderived material, inositol hexakisphosphate for human–machine interactions.^42^ The supramolecular hydrogel was attached on the depilated dorsal skin of mice, and no obvious irritative sign appeared. The histological analyses indicate that the supramolecular hydrogel did not cause any structural change in the skin. SIG materials also should be unharmful and less toxic to the human body. Meanwhile, an IL, 1-ethyl-3-methylimidazolium bis(trifluoromethanesulfonyl)imide [EMIM][TFSI] is widely used for energy storage devices, electrochemical transistors, and SIGs due to its hydrophobicity, high ionic conductivity, and large potential window.^11,12^ However, the IL possesses toxicity, which may render it unsuitable for biomedical applications. Hwang et al, evaluated the IL toxicity to human skin models.^53^ Cytotoxicity tests were conducted on ILs with the [EMIM] cation paired with various anions, including [TFSI], hexafluorophosphate ([PF_6_]), tetrafluoroborate ([BF_4_]), and dicyanamide ([DCA]). The [TFSI] anion, in particular, demonstrated significant cytotoxicity toward human keratinocyte (HaCaT) and human fibroblast (Hs68) cell lines. Given that HaCaT and Hs68 are primary cellular components of human dermis, [EMIM][TFSI] may pose adverse effects on human skin. The IL cytotoxicity persisted even when cations were replaced with 1-butyl-1-methylpyrrolidinium [BMPY], tributylmethylammonium [TBA], and zinc ion [Zn]. Toxicity assessments of [EMIM][TFSI] and [EMIM][BMPY] were conducted using 3D reconstructed human epidermis (Keraskin) and 3D reconstituted human full skin model (Keraskin-FT) MTT assay, indicating that [EMIM][TFSI] and [EMIM][BMPY] caused damage to Keraskin. With the development of transient electronics, the applications of the SIG are expanding to outdoor, and environmental friendliness and biodegradability of SIG materials are crucial for environmental sensing. Conventional ILs involve not only cytotoxicity but also environmental harm,^54^ which may hinder the SIG applications for transient electronics. Recently, bioderived ILs and deep eutectic solvents (DESs) are developed using natural products or materials that present in human body.^55,56^ These bioderived ILs show low toxicity, biodegradability, and biocompatibility, making them promising candidates as SIG electrolytes for contact with human skin and outdoor use.^57,58^ The success of transient electronics for biomedical applications^59−61^ indicates that the transient behavior of SIGs can be an essential property for developing electrochemical devices with low invasion. Polymer design is necessary to degrade SIGs after the prescribed period. The application of SIGs in transient electronics requires degrading the entire device. Hence, material design is crucial to focus on the selection of biodegradable metals (iron, magnesium, molybdenum, and tungsten),^62−64^ insulators ([poly(1,8-octanediol-*co*-citrates)],^65^ and ester bond photo-cross-linked poly[octamethylene maleate(anhydride)citrate]).^66,67^ SIGs exhibit favorable physical features such as toughness, self-healing capability, and self-adhesion for wearable devices. SIG integration with emerging materials, organic semiconductors,^68^ MXenes,^69,70^ or nanomaterials^71−73^ would produce sensors, transistors, or energy storage devices with excellent mechanical robustness, stability, and long-term operation. The rapid advancement in SIGs can achieve biomedical, wearable, and transient devices that involve excellent affinity to the human body.

**Table 1:** Comparison of Materials, Maximum Fracture Stress, Strain, Young’s Modulus, Adhesion Strength, and Self-Healing Efficiency from Recent Studies on SIGs

no.	materials (IL or DES/polymer)	maximum fracture strain (%)	maximum fracture stress (MPa)	maximum Young’s modulus (MPa)	maximum adhesion strength	self-healing efficiency (%)	year	ref
1	[Bmim][M_*x*_Br_*y*_ ] (M = Zn, Ca, Fe, or Cu)/PVA	5248 ± 113	63.1 ± 2.1	49.5 ± 1.4			2023	(25)
2	citric acid and l-(−)-carnitine/PDMAA	4900	14.4	48	24.4 MPa (glass)	∼90 (in 10 min at 25 °C, strain)	2024	(26)
3	[EMIM][TFSI]/P(DMAAm-r-AAc)	400	0.32			∼100 (in 3 h, strain)	2017	(27)
4	[EMIM][TFSI]/(P[AA][TFSI]-r-PHEA)	1130	∼0.50	0.84		∼90.3, ∼ 98.7, and ∼99.7 (in 1, 3, and 5 min respectively, at 25 °C, toughness)	2022	(28)
5	[ChCl][PDO]/HSAH and PHEAA	4000	0.26		13.92 ± 0.77 kPa (glass)	∼60 (at room temperature, extension)	2021	(29)
6	[EMI][TFSI]/PtBS and poly(MA-r-4HS) or poly(MA-r-2VP)	886 ± 168	0.14	15.62 ± 0.82	120 kPa (aluminum)		2021	(30)

## Data Availability

No new data were generated or analyzed as part of this perspective.
